# The Hypercomplex Genome of an Insect Reproductive Parasite Highlights the Importance of Lateral Gene Transfer in Symbiont Biology

**DOI:** 10.1128/mBio.02590-19

**Published:** 2020-03-24

**Authors:** Crystal L. Frost, Stefanos Siozios, Pol Nadal-Jimenez, Michael A. Brockhurst, Kayla C. King, Alistair C. Darby, Gregory D. D. Hurst

**Affiliations:** a Institute of Integrative Biology, University of Liverpool, Liverpool, United Kingdom; b Department of Animal and Plant Sciences, University of Sheffield, Sheffield, United Kingdom; c Department of Zoology, University of Oxford, Oxford, United Kingdom; Max Planck Institute for Marine Microbiology

**Keywords:** bacteriophage evolution, endosymbionts, genomics, plasmids

## Abstract

Mobile elements—plasmids and phages—are important components of microbial function and evolution via traits that they encode and their capacity to shuttle genetic material between species. We here report the unusually rich array of mobile elements within the genome of Arsenophonus nasoniae, the son-killer symbiont of the parasitic wasp Nasonia vitripennis. This microbe’s genome has the highest prophage complement reported to date, with over 50 genomic regions that represent either intact or degraded phage material. Moreover, the genome is predicted to include 17 extrachromosomal genetic elements, which carry many genes predicted to be important at the microbe-host interface, derived from a diverse assemblage of insect-associated gammaproteobacteria. In our system, this diversity was previously masked by repetitive mobile elements that broke the assembly derived from short reads. These findings suggest that other complex bacterial genomes will be revealed in the era of long-read sequencing.

## Abstract (precis)

The biology of many bacteria is critically dependent on genes carried on plasmid and phage mobile elements. These elements shuttle between microbial species, thus providing an important source of biological innovation across taxa. It has recently been recognized that mobile elements are also important in symbiotic bacteria, which form long-lasting interactions with their host. In this study, we report a bacterial symbiont genome that carries a highly complex array of these elements. Arsenophonus nasoniae is the son-killer microbe of the parasitic wasp Nasonia vitripennis and exists with the wasp throughout its life cycle. We completed its genome with the aid of recently developed long-read technology. This assembly contained over 50 chromosomal regions of phage origin and 17 extrachromosomal elements within the genome, encoding many important traits at the host-microbe interface. Thus, the biology of this symbiont is enabled by a complex array of mobile elements.

## OBSERVATION

Phages and plasmids play important roles in the ecology and evolution of bacteria (1). Both types of elements have the capacity to promote the lateral transfer of traits. Temperate phages commonly carry genes that encode phenotypes of benefit to bacterial survival, including key determinants of the ability to thrive in association with hosts (2–4). Plasmids are also key determinants of phenotype, most importantly acting as shuttles for antibiotic resistance (5). Plasmids also carry a range of traits important for association with hosts. For instance, both essential amino acid synthesis in the aphid symbiont *Buchnera* and the reproductive parasitic phenotype of male-killing *Spiroplasma* in Drosophila melanogaster are encoded on plasmids (6–7).

Recent advances in long-read sequencing technologies (read lengths of >20 kb) allow closure of prophage-rich bacterial genomes whose repetitive nature prevented them from being completed with previous technologies. Our own attempts to complete the genome of Arsenophonus nasoniae, the male-killing endosymbiont of *Nasonia* and other chalcid wasps (8), represents an instructive case study. This wasp species parasitizes filth fly (calliphorid) pupae. Arsenophonus nasoniae passes from a female into the fly pupa on wasp oviposition, from where it infects larvae through feeding, ultimately establishing as an extracellular infection in the wasp ovipositor (9). The microbe kills male hosts as embryos and may also exhibit pathological relationship in diapausing (overwintering) wasp larvae.

Our first attempt to sequence this genome used standard and paired-end libraries with 454 sequencing, resulting in a fragmented assembly with 665 contigs (143 scaffolds and 261 sequencing gaps) (10). We have since used Illumina, PacBio, and, lately, Oxford Nanopore reads to produce an improved reference genome assembly for A. nasoniae (strain FIN). Hybrid assembly using PacBio (15- to 20-kb-size-selected library, ∼4-kb median read length, ∼12× coverage) and Nanopore long reads (∼10-kb median read length, 252-kb maximum read length, 9,631 reads of >20 kb, ∼169× coverage) with subsequent Illumina polishing resulted in a closed circular genome (for details of strain isolation and sequencing methods, see https://doi.org/10.6084/m9.figshare.11842425).

**FIG 1:**
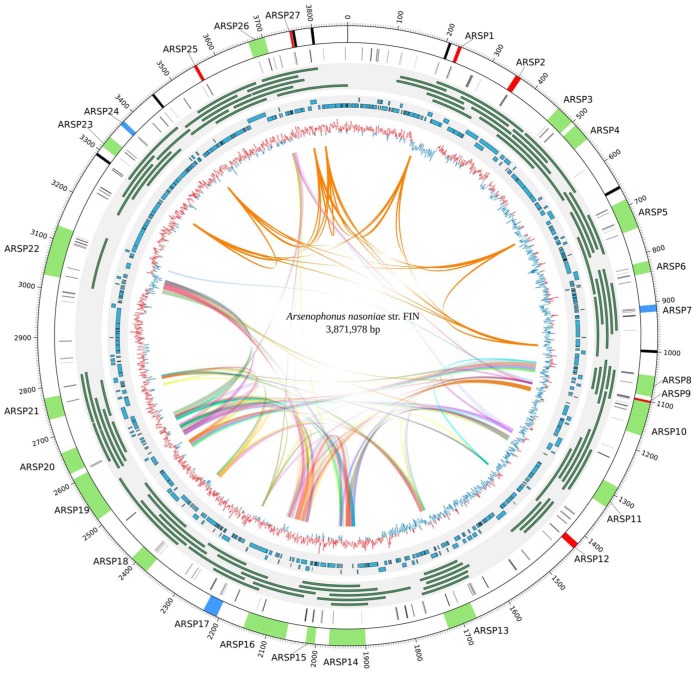
Prophage-rich content of *A. nasoniae* strain FIN’13’s main chromosome. Inwards, the first circle represents the complete and closed chromosome generated from the hybrid assembly of the Nanopore, PacBio, and Illumina data. The different colors depict intact (green), questionable (blue), and incomplete (red) phage elements, as estimated by PHASTER, while black bars represent the seven rRNA operons. The second circle depicts the transposable elements identified in the *A. nasoniae* genome. The third circle shows uniquely mapped long Nanopore reads spanning the prophage regions. Only the top five largest reads spanning each region are shown. The fourth circle shows the corresponding assembly using only the Illumina data. The innermost circle represents patterns of GC skew. Colored ribbons in the interior connect the different synteny blocks (>5 kb). The scale is in kilobase pairs.

This closed genome revealed a 3.9-Mb main chromosome with abundant phage-derived chromosomal islands (Fig. 1). PHASTER (11) estimates the presence of 27 phage-derived regions, of which 18 are classed as complete and may represent intact prophage, 3 are classed as unsure, and 6 are classed as incomplete and probable relics. The sizes of these chromosomal phage-derived regions range between 4.3 and 101.7 kbp (Fig. 1; see also https://doi.org/10.6084/m9.figshare.11845134). This density of phage-derived elements (6.9/Mb) is almost double that of Lactococcus lactis subsp. *cremoris* MG1363, the most prophage-rich genome recorded in previous systematic analyses (12). Of the 27 phage-derived elements inferred in the assembly, 26 are confirmed through individual reads that cover both flanking regions. All were confirmed through tiled long reads with unique overlapping sequences (Fig. 1).

Phage-derived elements classed as potentially complete prophage by PHASTER were examined in more detail (https://doi.org/10.6084/m9.figshare.11845182). Flanking attachment sites were observed in all cases. However, these putative prophage elements varied in their ranges of core phage functions identified through homology. Two of 18 putative prophage elements lacked genes predicted to function in lysis, 4 lacked predicted packaging genes, and 3 lacked structural genes. From these data, we conclude either (i) that some of these elements are not autonomous (excision would require complementation by other phage-derived elements) or (ii) that the missing components are novel and within the unannotated portion of the putative prophage element.

Synteny mapping using Sibelia (13) shows that the phage-derived regions within the *A. nasoniae* main chromosome are not identical but do share many repetitive elements (Fig. 1). This mosaicism of repetitive sequences breaks the assembly when the read length is insufficient to fully span the repetitive region(s) in any given phage-derived region, and these elements are the primary cause of assembly breakage in previous sequencing efforts, with break points commonly occurring in phage-derived regions.

**FIG 2:**
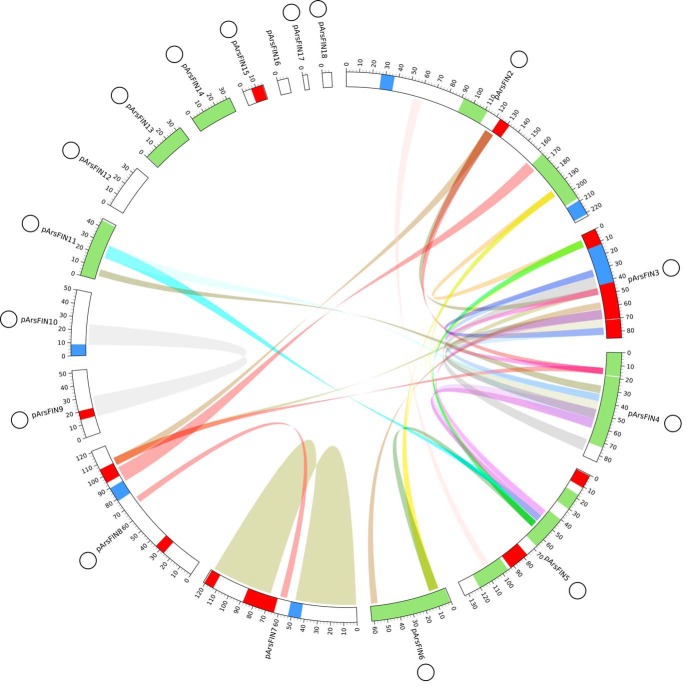
Plasmid maps of *A*. *nasoniae* in linearized form. Predicted intact, questionable, and incomplete prophage elements are shown in green, blue, and red, respectively. Colored ribbons connect the different synteny blocks (>5 kb). The assignation O indicates that the element is predicted to be circular. The scale is in kilobase pairs.

The assembly also predicted a complex complement of extrachromosomal DNA, with at least 17 extrachromosomal DNA elements (15 circular, 1 linear, 1 unclear) (Fig. 2). Patterns of coverage (https://doi.org/10.6084/m9.figshare.11845263), combinations of long-read pairs that overlap and form a circle, and the presence of open reading frames (ORFs) predicted to function in plasmid maintenance (retention systems and/or replication initiation proteins) support their status as plasmids (https://doi.org/10.6084/m9.figshare.11845197 and https://doi.org/10.6084/m9.figshare.11845305). The predicted plasmid diversity exceeds the 11 recorded in Marinovum algicola, which the authors considered “unprecedented for proteobacteria” (14). Outside of the proteobacteria, a similar number of extrachromosomal elements (up to 21) was found in Borrelia burgdorferi (15). This parasitic spirochete, like *A. nasoniae* (7), has two different host species in its life cycle (15). There are 1,426 predicted genes on the plasmids, of which 320 code for accessory genes and 419 are annotated as hypothetical (https://doi.org/10.6084/m9.figshare.11857836). ORFs present are predicted to encode diverse functions, including the capacity to induce apoptosis in host cells, toxin elements and transporters, type III secreted effectors that manipulate eukaryotic cell physiology, and proteins that allow microbes to adhere to and invade eukaryotic cells.

The extrachromosomal DNA is predicted to harbor 28 further phage-derived elements (PHASTER, 11 complete, 5 unsure, and 12 incomplete). Four of the extrachromosomal elements consisted entirely of circularized prophage DNA (https://doi.org/10.6084/m9.figshare.11845305) and were classified as plasmids on the basis of their Rep-encoding genes or addiction systems. Synteny mapping indicates that extrachromosomal elements share many repetitive elements with each other, and these regions are primarily of phage origin (Fig. 2). In contrast, only one 5-kb window from the extrachromosomal genome compartment was observed to show sequence similarity to the main chromosome (with a threshold of 90% of the nucleotide sequence) (https://doi.org/10.6084/m9.figshare.11857794).

Arsenophonus nasoniae represents the most-prophage-rich genome documented to date. With 55 predicted phage-derived regions in total, 27% of the main chromosome and 43% of the total genome are phage derived. Phage-derived regions on the main chromosome are predicted to encode 1,490 protein sequences. Of the genes, around 250 are not associated with core phage functions, and an additional 491 genes were annotated as encoding hypothetical proteins that lack domains recognized by Pfam. ORFs present are predicted to encode diverse functions, including the capacity to induce apoptosis in host cells, toxin elements and transporters, type III secreted effectors that manipulate eukaryotic cell physiology, and proteins that allow microbes to adhere to and invade eukaryotic cells (https://doi.org/10.6084/m9.figshare.11857836). However, they do not contain ORFs of obvious eukaryotic origin, as typified in the eukaryote association modules of *Wolbachia* phage (16).

The ORFs present in phage-derived regions highlight the potentially rich capacity for phages to drive lateral transfer of important genes between bacterial lineages. Previous work indicated shared phage-derived elements between *Arsenophonus* spp. and the aphid symbiont Hamiltonella defensa (17). We examined likely sources of the phage-derived elements of Arsenophonus nasoniae using Krona charts, examining the closest allied matches to ORFs within phage-derived regions (https://doi.org/10.6084/m9.figshare.11857860). Of the genes in *A. nasoniae* phage-derived regions, the best matches were in the genus *Arsenophonus*, other closely related genera, and other insect-associated gammaproteobacteria. In contrast, with regard to the genes not present within the predicted phage-derived regions, the best matches were most commonly to genes in the *Arsenophonus* symbiont from Nilaparvata lugens.

We complemented this approach with phylogenetic analysis of three prophage-encoded proteins: portal protein, lysozyme, and capsid protein (https://doi.org/10.6084/m9.figshare.11857869). Predicted capsid proteins and predicted phage portal proteins from *A. nasoniae* prophage-derived elements were most closely related to the respective gene in prophage elements from *Xenorhabdus*, *Proteus*, *Providencia*, and *Morganella*, genera closely related to *Arsenophonus*. In contrast, predicted lysozyme proteins from *A. nasoniae* prophage-derived regions had a distinct pattern, with most closely related genes being from other *Arsenophonus* strains, and this cluster then allied to ones derived from prophages in the aphid symbiont Regiella insecticola.

Overall, there is no consistent pattern supporting direct lateral transfer of an intact phage element from any currently sampled taxon. Rather, the elements observed are chimeric, predominantly with nearest matches from closely allied genera, but with some components deriving from more distantly related insect-associated gammaproteobacteria. Past work examining phage transfer in *Wolbachia* has emphasized the importance of coinfection as an arena for transfer (18). As a gammaproteobacterium that exists both as an endosymbiont and external to hosts, *Arsenophonus* likely encounters a broad range of microbes, and this establishes opportunities for lateral transfer. Arsenophonus nasoniae, for instance, exists alongside both *Proteus* and *Providencia* within the *Nasonia* gut (19).

Phage transfers have occurred despite the presence of a type I CRISPR-Cas system in the *A. nasoniae* genome. The main chromosome of *A. nasoniae* carries a CRISPR-Cas system most similar in organization to that found in Yersinia pseudotuberculosis (type I-F) (https://doi.org/10.6084/m9.figshare.11857887). A notable difference is that in *A. nasoniae*, the *cas2-cas3* fusion gene, a typical characteristic of the type I-F CRISPR-Cas system (20), is interrupted by a degenerated insertion element of the IS*91* family and by a small gene predicted to encode a hypothetical protein. Diverse spacer elements are found adjacent to the CRISPR-Cas cluster. Eight of the 11 spacers show homology to either prophage or plasmid sequences found in the *A. nasoniae* genome; the other three are of unknown origin and potentially evidence elements that were historically present. It is possible that the CRISPR system suppresses prophage reactivation in *A. nasoniae*, as observed in other systems (21). It is currently unclear if the intact prophage elements retain the capacity to establish a lytic cycle.

This genome was challenging to assemble because of its structural complexity. It is notable that many of the reported closed bacterial chromosomes with high predicted prophage content were obtained using methods developed prior to next-generation sequencing (NGS), in which cosmid- or fosmid-based scaffolding processes are employed (https://doi.org/10.6084/m9.figshare.11857893). These long-range scaffolding tools make assemblies relatively robust for long repetitive elements, such as prophages. Conversely, closed bacterial chromosomes completed using short-read sequencing methods tend not to contain large numbers of phage-derived elements. Short-read sequencing methods may have thus led to an underrepresentation of closed bacterial genomes with complex architectures. Because the causes of assembly failure (repetitive elements like phages [22]) are biologically important, these broken assemblies obscure important properties. A full understanding of the role of prophages in the genome evolution of bacteria will require more widespread application of long-read sequencing. Without this approach, we will continue to underestimate the complexity and diversity of host-associated microbes.

### Data availability.

Raw sequences have been submitted to the NCBI SRA under accession numbers SRR8797496, SRR8797497, and SRR8797498 for PacBio, Illumina, and Nanopore data, respectively. Data can be found under BioProject number PRJNA529362. Supplementary material may be downloaded from https://doi.org/10.6084/m9.figshare.c.4853367.v1.
